# Increased extravascular lung water index (EVLWI) reflects rapid non-cardiogenic oedema and mortality in COVID-19 associated ARDS

**DOI:** 10.1038/s41598-021-91043-3

**Published:** 2021-06-01

**Authors:** Sebastian Rasch, Paul Schmidle, Sengül Sancak, Alexander Herner, Christina Huberle, Dominik Schulz, Ulrich Mayr, Jochen Schneider, Christoph D. Spinner, Fabian Geisler, Roland M. Schmid, Tobias Lahmer, Wolfgang Huber

**Affiliations:** 1 Department of Internal Medicine II, School of Medicine, University Hospital Rechts Der Isar, Technical University of Munich, Ismaninger Straße 22, 81675 Munich, Germany; 2 Department of Dermatology and Allergology, School of Medicine, Technical University of Munich, Biedersteiner Str. 29, 80802 Munich, Germany; 3 German Center for Infection Research (DZIF), Partner Site Munich, Munich, Germany

**Keywords:** Viral infection, Respiratory distress syndrome

## Abstract

Nearly 5% of patients suffering from COVID-19 develop acute respiratory distress syndrome (ARDS). Extravascular lung water index (EVLWI) is a marker of pulmonary oedema which is associated with mortality in ARDS. In this study, we evaluate whether EVLWI is higher in patients with COVID-19 associated ARDS as compared to COVID-19 negative, ventilated patients with ARDS and whether EVLWI has the potential to monitor disease progression. EVLWI and cardiac function were monitored by transpulmonary thermodilution in 25 patients with COVID-19 ARDS subsequent to intubation and compared to a control group of 49 non-COVID-19 ARDS patients. At intubation, EVLWI was noticeably elevated and significantly higher in COVID-19 patients than in the control group (17 (11–38) vs. 11 (6–26) mL/kg; p < 0.001). High pulmonary vascular permeability index values (2.9 (1.0–5.2) versus 1.9 (1.0–5.2); p = 0.003) suggested a non-cardiogenic pulmonary oedema. By contrast, the cardiac parameters SVI, GEF and GEDVI were comparable in both cohorts. High EVLWI values were associated with viral persistence, prolonged intensive care treatment and in-hospital mortality (23.2 ± 6.7% vs. 30.3 ± 6.0%, p = 0.025). Also, EVLWI showed a significant between-subjects (r = − 0.60; p = 0.001) and within-subjects correlation (r = − 0.27; p = 0.028) to Horowitz index. Compared to non COVID-19 ARDS, COVID-19 results in markedly elevated EVLWI-values in patients with ARDS. High EVLWI reflects a non-cardiogenic pulmonary oedema in COVID-19 ARDS and could serve as parameter to monitor ARDS progression on ICU.

## Introduction

COVID-19 is caused by Severe Acute Respiratory Coronavirus 2 (SARS-CoV-2) and shows a wide clinical spectrum covering asymptomatic cases, mild upper respiratory affectation, and severe pneumonia^1,2^. While the majority of patients have a favorable outcome, higher age and underlying comorbidities are associated with a poor prognosis. Typically, patients with severe COVID-19 pneumonia suffer from dyspnoea, hypoxemia, massive alveolar damage, progression to acute respiratory distress syndrome (ARDS) and multiple organ failure^3^.

The pathogenesis of COVID-19 is poorly understood. As far as known, onset of COVID-19 associated ARDS leads to uncontrolled pulmonary inflammation, fluid accumulation, and progressive fibrosis that severely compromise oxygen and carbon dioxide exchange^4^. Moreover, it is assumed that a complex immune response of the host to the SARS-CoV-2 virus results in an uncontrolled release of inflammatory proteins^5–8^.

Regarding the predominantly higher age and a substantial prevalence of circulatory comorbidities such as coronary heart disease, peripheral artery disease, arterial hypertension and diabetes mellitus, the role of cardiogenic implications on pulmonary oedema has to be further studied^2,3,5,7^.

Single indicator transpulmonary thermodilution (TPTD) is a commercially available technology of advanced hemodynamic monitoring. TPTD provides bedside measurement of extravascular lung water index (EVLWI) which is a marker of pulmonary oedema. Additionally, crucial hemodynamic parameters such as stroke volume index (SVI), global ejection fraction (GEF) and the preload marker global end-diastolic volume index (GEDVI) are derived from TPTD^9–11^.

Several studies demonstrated significant and independent association of EVLWI and its changes over time with mortality in ARDS^12–17^. A recent study found EVLWI among the best markers to improve early prediction of 28-days-mortality in patients with non-COVID-19 ARDS compared to traditional scores of ARDS severity^18^. Furthermore, TPTD-monitoring of critically ill patients with non-COVID-19 ARDS was independently associated with a lower mortality in this study.

To date, data on hemodynamic key parameters generated by bedside TPTD, especially on EVLWI, are lacking in COVID-19-patients.

Primary objective of this study is to investigate EVLWI in the context of other key hemodynamic and pulmonary parameters derived from TPTD in mechanically ventilated patients with COVID-19 ARDS compared to a recent cohort with non-COVID-19 ARDS. In addition, we evaluate the potential of EVLWI to predict outcome and monitor ARDS progression in patients with severe COVID-19.

## Material and methods

The study protocol was approved by the Institutional Review Board (Ethics committee of Technical University of Munich; Approval No. 178/20S) as part of the register study CORRECT: COVID Registry REChts der Isar intensive care Trial. The study was registered at the Clinical Trial Registry (ISRCTN10077335) and all methods were performed in accordance with the relevant guidelines and regulations. Additional data of the study and control group is reported in supplementary table 1.

All patients or their legal representatives gave written informed consent. The study was conducted in a COVID-19-ICU with 14 beds at the tertiary referral hospital Klinikum rechts der Isar in March and April 2020.

### Inclusion and exclusion criteria

All patients were diagnosed with COVID-19 (confirmed by PCR), intubated, mechanically ventilated, and suffered from ARDS, according to the Berlin definition^19^. Patients were excluded if TPTD was contra-indicated (lower extremity peripheral artery disease grade II or above according to the Forestier classification) or not feasible within the first 12 h after intubation. Patients receiving other vasopressors than norepinephrine were also excluded. Since extracorporeal membrane oxygenation (ECMO) might lead to incorrect measurement of EVLWI and GEDVI, TPTD measurements during ECMO therapy were not included^20^.

According to the local standard, TPTD was performed at least once within 24 h as described previously^11,21^.

In brief a, 5F thermistor-tipped arterial line (PV2025L20, Pulsiocath, Pulsion Medical Systems, SE Feldkirchen Germany) was inserted into the femoral artery. The thermistor line and the pressure line of the arterial catheter as well as a second thermistor on the central venous catheter (CVC) for measurement of the injectate temperature were connected to a hemodynamic monitor (PiCCO-2 or PulsioFlex, both Pulsion Medical Systems, SE Feldkirchen Germany). The TPTD curve was registered and analyzed after injection of 15 mL icecold 0.9% saline solution via CVC. Each TPTD value represents the mean of three consecutive thermodilution measurements within 5 min.

EVLWI was indexed to predicted bodyweight as suggested by the manufacturer^22^.

To derive EVLWI, GEDVI, SVI, GEF and all other parameters provided by the PiCCO, we used the most recent software V3.1, which corrects GEDVI for femoral CVC indicator injection^23^. High pulmonary vascular permeability index (PVPI) values (≥ 3) are associated with inflammation and pulmonary origin, whereas low values indicate cardiogenic or mixed pulmonary oedema. PVPI is calculated as a ratio from unindexed extravascular lung water EVLW divided by pulmonary blood volume (PBV). PBV is assumed to be about 25% of unindexed GEDV (PVPI = EVLW/(0.25*GEDV))^24^. Since the correction for femoral CVC placement does not pertain to PVPI, PVPI derived from femoral indicator injection (PVPI_fem) was corrected in both cohorts as suggested recently^24^.

Correction is based on two formulas:

PVPI_fem_corrected = PVPI_fem * GEDVI_fem_uncorrected/GEDVI_fem_corrected and.

GEDVI_fem_corrected = 0.539 *GEDVI_fem_uncorrected − 15.15 + 24.49 *CI_fem + 2.311*IBW^23,24^.

PVPI_fem, GEDVI_fem and CI_fem: PVPI, GEDVI and PVPI derived from femoral indicator injection. IBW: Ideal bodyweight. See Huber et al. for further details^14^.

For this analysis TPTD and respiratory parameters were routinely registered in included all patients on ICU at intubation and on day 3,7,10 and 14. To record ARDS severity we calculated Horowitz index (PaO2/FiO2 ratio) at the time of TPTD measurement.

The control group consists of 49 consecutive patients with TPTD monitoring and non-COVID-19 ARDS^19^. All patients of this cohort were treated in the same ICU as the COVID-19 patients before 2019 (see Clinical Study Registration No. ISRCTN32938630; Institutional Review Board (Ethics committee of Technical University of Munich), Approval No. 343/18 S).

### Primary endpoint

Comparison of EVLWI at admission and during treatment on ICU in COVID-19 ARDS patients with a recent cohort with non-COVID-19 ARDS^18^.

### Secondary endpoints

- EVLWI as potential parameter to monitor ARDS progression and predict in-hospital mortality

PVPI, SVI, GEF and GEDVI in COVID-19 and non-COVID-19 ARDS patients

### Power calculation

Based on two independent study groups, a continuous endpoint (EVLWI with a mean of 12.5 ± 4.9 mL/kg in the non-COVID-19 cohort and an estimated EVLWI of 18 ± 7 mL/kg in the COVID-19 cohort), a number of 49 non-COVID 19 and 25 COVID-19 patients would result in a statistical power of > 90% with a p-value of p < 0.05^18^.

### Statistics

Statistical analysis was performed using IBM SPSS Statistics 25 (SPSS Inc, Chicago, Illinois, USA). Samples were checked for normal distribution using the Shapiro–Wilk test. Descriptive data of normally distributed parameters are presented as mean ± standard deviation and as median and range for non-parametric parameters. The Mann–Whitney-U and Kruskal–Wallis tests were used to analyze non-parametric variables and the t-test as well as a one-way analysis of variances (ANOVA) to analyze variables with normal distribution. To compare qualitative parameters, chi-square test and in small samples (expected frequency of test variable less than 5) Fisher's exact test was used. All statistical tests were two-sided, p-values of < 0.05 were considered significant. Multivariate linear regression models were used to identify parameters that are independently associated with higher EVLWI and PVPI values. Factors with a significant p-value below 0.05 in univariate analysis were included in the regression models. Each variables impact in the regression model is reported by the coefficient beta. To control the false discovery rate after multiple testing, we adjusted the level of significance by the Benjamini–Hochberg procedure. Spearman’s p was used for nonparametric rank correlation. To assess whether ELVWI can be used to monitor respiratory function and ARDS progression over time we calculated between-subject and within-subject correlations to Horowitz index as proposed by Bland et al.^25,26^.

## Results

In total, 74 patients with ARDS were included in the study (25 with COVID-19 and 49 without). Patient characteristics are shown in Table 1.

**Table 1:** Patient characteristics.

Parameter	COVID-19 ARDS patients (n = 25)	Non-COVID-19 ARDS patients (n = 49)	p-value (adj. p = 0.02)
Age [years]	68 (35–84)	65 (23–87)	p = 0.815
Gender [male/female]	21/5	26/23	**p = 0.018**
BMI [kg/m2]	24.8 (18.5–49.0)	24.7 (17.3–37.0)	p = 0.416
SOFA-score	6 (3–13)	12 (2–21)	**p < 0.001**
APACHE-II	12 ± 5	22 ± 8	**p < 0.001**
Neutrophil / lymphocyte ratio at intubation	9.44 (3.24 – 94)		
Days on ICU	22.9 ± 8.6		
Days on mechanical ventilation	13.5 (2–55)	10.0 (1–28)	p = 0.119
SARS-CoV-2 clearance	22/26 (84.6%)		
Days till SARS-CoV-2 clearance	20.0 ± 8.6		
Mortality	9/26 (34.6%)	16/49 (32.7%)	p = 0.864

*BMI* body mass index, *ICU* intensive care unit, *adj. p* p value adjusted for multiple testing, significant p-values are displayed in bold letters.

### Biometric data and scores

Patients with COVID-19 were more frequently male compared to non-COVID-19 patients (20/25 (80%) vs. 26/49 (53%); p = 0.041; Table 1). SOFA and APACHE-II-score were higher in the non-COVID-19 group.

### Respiratory data

Summarizing several of the respiratory parameters (reported in Table 2), the oxygenation index (OI = Paw_mean * FiO_2_/pO_2_) was 66% higher in the COVID-19 cohort (14.1 ± 9.9 vs. 8.5 ± 4.4; p = 0.005).

**Table 2:** Respiratory baseline parameters.

Parameter	COVID-19 ARDS patients (n = 25)	Non-COVID-19 ARDS patients (n = 49)	p-value
P_peak	26 (20–40)	27.5 (12–32)	p = 0.631
PEEP [cm H_2_O]	14 (5–20)	8 (6–15)	**p < 0.001**
Tidal volume [mL/kg]	6.58 ± 1.65	6.67 ± 2.18	p = 0.863
pO_2_/FiO_2_ (Horovitz-index)	148 ± 81	187 ± 62	**p = 0.041**
OI	14.1 ± 9.9	8.5 ± 4.4	**p = 0.005**
**ARDS Berlin-definition**			
Mild	5/26 (19%)	22/49 (45%)	p = 0.028*
Moderate	16/26 (62%)	24/49 (49%)	
Severe	5/26 (19%)	3/49 (6%)	

* mild vs. moderate/severe, *P_peak* maximal inspiratory pressure, *PEEP* positive end-expiratory pressure, *pO*_*2*_ partial pressure of oxygen, *OI* oxygenation index, *ARDS* acute respiratory distress syndrome, tidal volume is reported in ml/kg predicted body weight, significant p-values are displayed in bold letters.

### Parameters derived from TPTD and pulse contour analysis (PCA)

TPTD data are reported in Table 3.

**Table 3:** Initial (at intubation) measurement of hemodynamic data.

Parameter	COVID-19 ARDS patients (n = 25)	Non-COVID-19 ARDS patients (n = 49)	p-value (adj. p = 0.025)
Heart rate [/min]	82 ± 21	99 ± 20	**p = 0.001**
MAP [mmHg]	78 ± 9	79 ± 16	p = 0.809
dPmax	1100 (531–2300)	1251 (580–2629)	p = 0.176
GEDVI	761 ± 148	746 ± 180	p = 0.829
EVLWI	17 (11–38)	11 (6–26)	**p < 0.001**
SVI	38 ± 16	42 ± 16	p = 0.314
CI	3.0 (1.6–10)	3.7 (1.4–9.3)	**p = 0.008**
PVPI	2.9 (1.0–5.2)	1.9 (1.0–5.2)	**p = 0.003**
PVPI > 3	13/26 (50%)	8/49 (16.3%)	**p = 0.002**
Norepinephrine [µg/h]	400 (0–2400)	800 (50–8000)	p = 0.072

*MAP* mean arterial pressure, *dPmax* cardiac contractility index, *GEDVI* global end-diastolic volume index, *EVLWI* extra vascular lung water index, *SVI* stroke volume index, *CI* cardiac index, *PVPI* pulmonary vascular permeability index, *adj. p* p value adjusted for multiple testing.

### EVLWI and pulmonary vascular permeability index (PVPI) in COVID-19 patients versus non-COVID-19 patients

**Figure 1:**
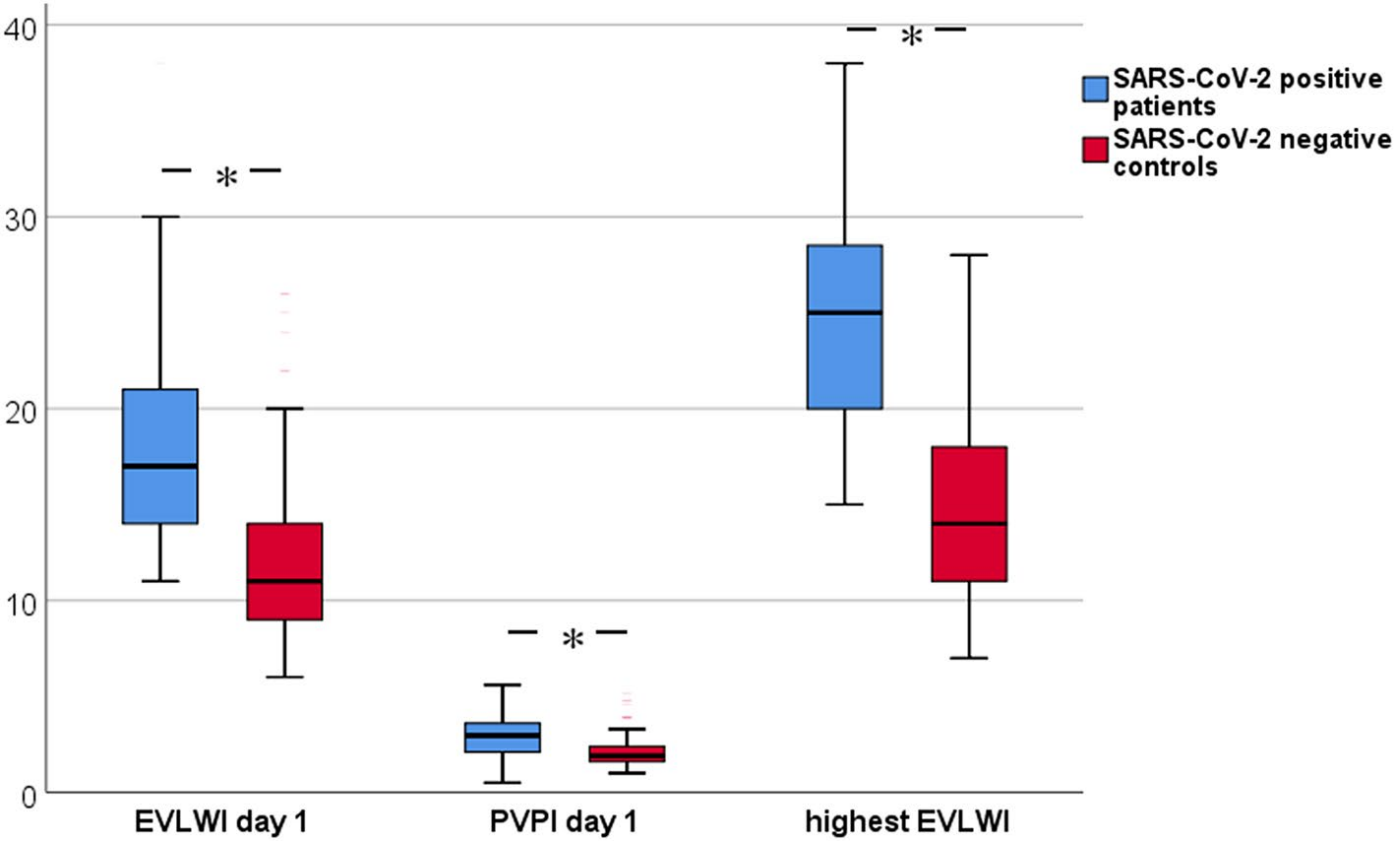
Boxplots comparing extra vascular lung water index (EVLWI) and pulmonary vascular permeability index (PVPI) on day 1 and highest EVLWI between patients with and without COVID-19; * indicates significance with p < 0.001.

EVLWI on day-1 (the day of intubation) and the highest EVLWI within the first 14 days after intubation (25.0 (15.0–43.0) vs. 14.0 (7.0–54.0); p < 0.001) were substantially higher in COVID-19 patients vs. non-COVID-19 patients (Table 3; Fig. 1 boxplots).

PVPI was significantly higher in patients with COVID-19 compared to the non-COVID-19 cohort on day-1 (Table 2 and Fig. 1).

In univariate analysis, the initial EVLWI was associated with COVID-19 (r = 0.503; p < 0.001) and there was a weak correlation with low body mass index (BMI) (r = − 0.264; p = 0.035), but not with gender, height, age, heart rate, MAP, SVRI, CVP, GEDVI, dPmax, SVI, CI, CPI or norepinephrine dosage.

Multivariable regression analysis (r = 0.508; R^2^ = 0.258) regarding EVLWI including COVID-19 status and BMI demonstrated that both COVID-19 (p < 0.001, Beta = − 0.494) and BMI (p = 0.03, Beta = − 0.228) were independently associated with higher EVLWI values.

PVPI was univariately associated with COVID-19, low BMI (r = − 0.338; p = 0.003) and low SVI (r = − 0.230; p = 0.048), but not with gender, height, age, heart rate, MAP, SVRI, CVP, dPmax, SVI, GEF, CPI or norepinephrine dosage.

In multivariable analysis (r = 0.519; R^2^ = 0.269), PVPI was independently associated with COVID-19 (p = 0.001; Beta = − 0.363) and low BMI (p = 0.001, Beta = − 0.355), but not with SVI. GEDVI and EVLWI were not included in the multivariable analysis regarding PVPI, since PVPI is derived from the ratio of unindexed EVLW divided by 0.25*GEDV.

**Figure 2:**
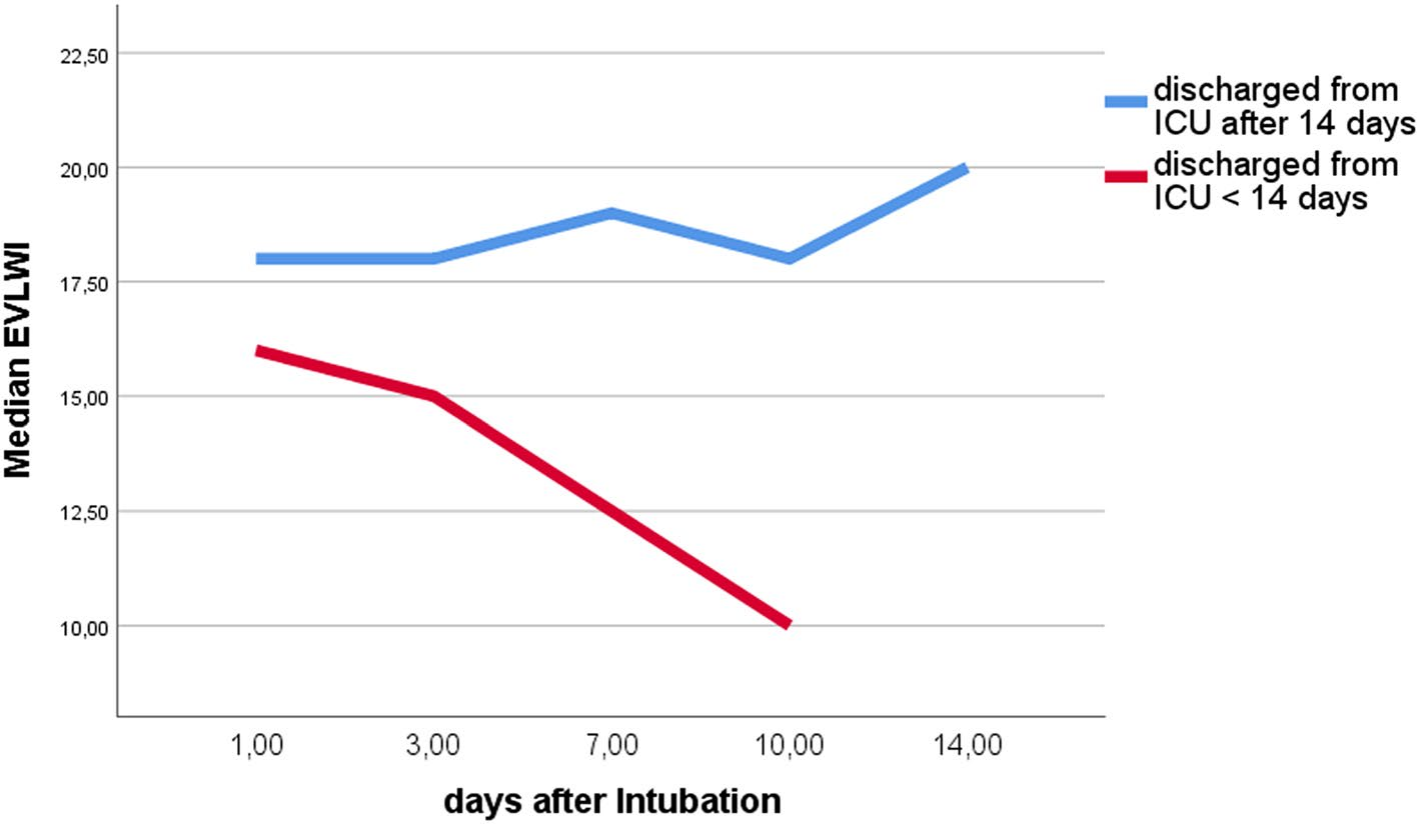
Extra vascular lung water index (EVLWI) of patients with COVID-19 who required less and more than 14 days of treatment on intensive care unit (ICU).

At intubation EVLWI correlated with OI (r = 0.58, p = 0.004). There was no significant correlation to the neutrophil/lymphozyte ratio. A significant EVLWI decrease during the first days of mechanical ventilation was associated with ICU treatment of less than 14 days and inversely associated with mortality (EVLWI at day 10 after intubation: 19.2 ± 7.5 vs. 10.0 ± 1.4, p = 0.002, Fig. 2; mortality: deltaEVLWI 7 (0–22) versus 3 (0–12), p = 0.021). There is a significant between- and within-subjects correlation of EVLWI and Horowitz index (r = − 0.60, p = 0.001 and r = − 0.27, p = 0.028).

Persistence of SARS-CoV-2 in respiratory samples of COVID-19 patients during the ICU stay was associated with mortality (17/17 vs. 5/9, p = 0.008). The highest EVLWI is associated with SARS-CoV-2 clearance (29.7 ± 2.5 vs. 24.6 ± 7.4, p = 0.046) and mortality (23.2 ± 6.7 vs. 30.3 ± 6.0, p = 0.025). The highest EVLWI was measured 5.2 ± 4.4 days after intubation in patients with COVID-19 ARDS.

### Preload markers GEDVI and CVP

By contrast, the static preload markers GEDVI (761 ± 168 vs. 749 ± 180 mL/m^2^; p = 0.882) and CVP (16.4 ± 7.4 vs. 17.9 ± 8.0 mmHg; p = 0.446) were not significantly different between ARDS patients with and without COVID-19.

### Parameters of cardiac function in COVID-19 patients versus controls

Global ejection fraction (GEF) (20.9 ± 6.0 vs. 23.8 ± 7.3%; p = 0.098), stroke volume index (SVI) and dPmax were comparable for patients with and without COVID-19 on day-1 (Table 2 and supplementary table 1).

## Discussion

This study demonstrates that EVLWI values are higher in patients with COVID-19 ARDS than in comparable patients with non-COVID-19 ARDS while there is no difference in TPTD parameters for cardiac function. In addition, a high EVLWI at intubation is associated with a prolonged need or intensive care treatment and increased mortality. During treatment changes in EVLWI correlate with severity of COVID-19 associated ARDS.

Severity of SARS-CoV-2 infections ranges from asymptomatic to severe ARDS. Similarly, some patients with COVID-19 associated ARDS recover within several days while others require mechanical ventilation for weeks or fail to recover at all. The reasons for this discrepancy are unclear and it is difficult to predict an individual patient’s prognosis. According to published data, a high EVLWI is associated with mortality in patients with ARDS^13–15^. We found a median EVLWI of 17 ml/kg, which is higher compared to both our previous non-COVID-19 ARDS cohort and previous studies performed in patients with non-COVID-19 ARDS^12,14–17^. The absolute, non-indexed EVLW for a 70 kg healthy patient would be around 500 mL. In non-COVID-19 ARDS patients, it is 900 mL with the best cut-off to predict increased mortality at 1000 mL^27^. In COVID-19 patients, EVLW reaches up to 2600 mL. Hence, there is no defined EVLWI cut-off for the prediction of mortality as absolute EVLWI values are not comparable between patients with COVID-19 ARDS and non-COVID-19 ARDS. While mortality is similar in both groups, EVLWI values differ significantly. In conclusion, within patients with COVID-19 high EVLWI values can predict mortality. In addition, the course of EVLWI values can help to monitor respiratory function of COVID-19 patients. Decreasing EVLWI values were associated with improved respiration and consequently less treatment days on ICU. Between-subjects correlation reveals a moderate to good correlation of EVLWI with Horowitz Index. Although weaker, within-subject correlation is also significant. Given these facts and considering its association with mortality, we think EVLWI is a good parameter to monitor ARDS progression in patients with COVID-19. Taking into account the within-subject correlation, EVLWI values have to be interpreted in the context of other clinical parameters, though.

The morphologic correlate of pronounced pulmonary inflammation appears as diffuse interstitial oedema on computed tomography (CT) that can affect large parts of the pulmonary tissue^28^. At intubation the comparatively high EVLWI values in COVID-19 patients correlate with a high OI as marker of lung injury^29^. Potentially, the degree of alveolar damage is the lung pathologic determinant of survival. But similar to non-COVID-19 ARDS this cannot be easily measured^30^. In patients that did not survive, a recent autopsy study reported pronounced endothelial damage and widespread capillary microthrombi in COVID-19 ARDS^31^. Similar to sepsis, a massive inflammatory response might explain this microangiopathy. In combination with intravascular coagulation and capillary leakage, this results in extensive pulmonary oedema. Lungs of COVID-19 patients with ARDS have a lower weight at autopsy compared to influenza associated ARDS, which seems contradictive to the increased EVLWI values. However, these two findings might be explained by the different time point when measurements were performed. EVLWI values were derived from the first days after intubation whereas autopsy is carried out later after termination of treatment.

In multivariable regression analysis EVLWI was negatively associated with BMI, whereas Giacomelli et al. suggest body weight to be associated with a bad outcome in COVID-19^32^. However, the negative correlation between EVLWI and BMI was very weak. There is data supporting an indexation to height rather than body weight as height increases EVLWI values and an EVLW indexed to height predicts FiO_2_/pO_2_ more accurately than an EVLW indexed to ideal body weight^14,22^. Increasing height results in lower BMI values which might cause the negative association of BMI and EVLWI.

In addition to the absolute increase in EVLWI, our study gives several hints that the COVID-19 related pulmonary oedema is mainly non-cardiogenic. The PiCCO-device combines TPTD with pulse contour analysis and provides a number of well-validated parameters of cardiac function. To facilitate decision support, a number of ratios is calculated, including PVPI and GEF (GEF = 4*stroke volume divided by GEDV).

PVPI relates EVLWI to preload (PVP = EVLW/(0.25*GEDV)). High PVPI values (in particular > 3) indicate pulmonary origin of the oedema with a normal GEDV. By contrast, elevated EVLWI-values in the context of a PVPI < 2 suggests cardiac dilatation with an elevated GEDVI. A PVPI of 3.1 ± 1.3 in our COVID-19 cohort suggests a non-cardiogenic pulmonary oedema.

This is further supported by GEF of 21 ± 6%, SVI of 38 ± 17 mL/m^2^ and dPmax of 1133 ± 402 mmHg/s. These parameters were comparable between COVID-19- and non-COVID-19 patients in our study. Mean values of GEF, SVI and dPmax were slightly below the normal range. However, reference ranges are given for a population with a representative age distribution. A recent study demonstrated that cardiac function as measured by cardiac output (CO) substantially decreases with older age (independent decrease of CO of 66 mL/min per year)^33^. Therefore, GEF, SVI and dPmax might be considered within the age-adjusted normal range.

Repeated CT scans are an alternative diagnostic tool to monitor inflammation and ARDS progression. However, inter-observer agreement depends on experienced staff and transport of ventilated patient always inherits a risk for the patient^34^. As demonstrated in our study, TPTD is a bedside available method to directly measure EVLWI with limited invasiveness in the ICU-setting. It has been well validated compared to the more invasive double-indicator technique^27,35,36^. EVLWI has not only the potential to predict mortality, but also to monitor ARDS and the extent of pulmonary oedema during intensive care treatment.

### Limitations of the study

As a single center study the results could be prone to a selection bias and confirmation of the reported findings in a larger multi-center cohort would be preferable.

Slight baseline differences of the biometric data from COVID-19 and non-COVID-19 cohorts can most likely be explained by older age and predominantly male gender in the COVID-19 cohort. Administration of intravenous fluids might influence TPTD parameters. However, as reflected in the different SOFA scores multi-organ failure was more frequent in the non-COVID-19 patients compared to a predominantly respiratory failure in the COVID-19 patients. So fluid administration would result in higher EVLWI values, especially in the non-COVID-19 cohort. Therefore, we do not think, that treatment with intravenous fluid has a relevant impact on our conclusions.

## Conclusion

EVLWI values in COVID-19 patients with ARDS are significantly higher than in non-COVID-19 ARDS patients. High EVLWI values are associated with increased mortality in patients with COVID-19 ARDS. Elevated EVLWI reflects a non-cardiogenic pulmonary oedema in COVID-19 associated ARDS and might serve as a parameter to monitor ARDS progression in ventilated patients on ICU.

## Supplementary Information

Supplementary Information.

## Data Availability

All data relevant for the analysis and conclusions of this study are included in this published article (and its Supplementary Information files). Exceeding information is available from the corresponding author on reasonable request.
