# Supplementary material for: Increased extravascular lung water index (EVLWI) reflects rapid non-cardiogenic oedema and mortality in COVID-19 associated ARDS
Source: Sci Rep. 2021 Jun 1;11:11524. doi: 10.1038/s41598-021-91043-3 (PMC8169693; doi:10.1038/s41598-021-91043-3)
Supplement: Supplementary file 1 — Supplementary Information. [file 41598_2021_91043_MOESM1_ESM.pdf]

Title: Increased extravascular lung water index (EVLWI) reflects rapid non-cardiogenic oedema and mortality in COVID-19 associated ARDS

Authors: Sebastian Rasch[1,]*, Paul Schmidle[2], Sengül Sancak[1], Alexander Herner[1], Christina Huberle[1], Dominik Schulz[1], Ulrich Mayr[1], Jochen Schneider[1], Christoph D. Spinner[1,3], Fabian Geisler[1], Roland M. Schmid[1], Tobias Lahmer[1,#], Wolfgang Huber[1,†,#]

Supplementary table 1: Additional respiratory and hemodynamic parameters

Parameter	COVID-19 patients	Non-COVID-19 patients	p-value
p_aO_2 [mmHg]	104 (47-360)	93 (58-178)	p=0.059
p_aCO_2 [mmHg]	42 (30-96)	41 (27-78)	p=0.727
Delta-pressure	15±4	18±4	p=0.009
P_mean	17±4	13±3	p<0.001
CVP [mmHg]	17±7	18±8	p=0.485
SVRI	1409 (400-3086)	1229 (400-3306)	p=0.076
CPI	0.48 (0.26-0.90)	0.60 (0.20-1.60)	p=0.009
